# Subsequent risk of acute urinary retention and androgen deprivation therapy in patients with prostate cancer: A population-based retrospective cohort study

**DOI:** 10.1097/MD.0000000000018842

**Published:** 2020-02-14

**Authors:** Teng-Kai Yang, Chia-Chang Wu, Chao-Hsiang Chang, Chih-Hsin Muo, Chao-Yuan Huang, Chi-Jung Chung

**Affiliations:** a Department of Surgery, Yonghe Cardinal Hospital; b School of Medicine, College of Medicine, Fu-Jen Catholic University; c Institute of Epidemiology and Preventive Medicine, College of Public Health, National Taiwan University; d Department of Urology, Shuang-Ho Hospital; e Department of Urology, School of Medicine, College of Medicine, Taipei Medical University; f Department of Urology; g Department of Medicine; h Management Office for Health Data; i Department of Urology, Taipei; j Department of Urology, National Taiwan University Hospital, Hsin Chu Branch, Hsin Chu City; k Department of Public Health, College of Public Health, China Medical University; l Department of Medical Research, China Medical University and Hospital, Taichung, Taiwan.

**Keywords:** acute urinary retention, androgen deprivation therapy, luteinizing hormone-releasing hormone, orchiectomy, prostate cancer

## Abstract

Acute urinary retention (AUR) is associated with hormone imbalance in men. However, limited studies focused on exploring the complications of AUR in patients with prostate cancer (PC) who receive androgen deprivation therapy (ADT). Therefore, we aim to evaluate the subsequent risk of AUR in ADT-treated PC patients. We collected data from 24,464 male patients who were newly diagnosed with prostate malignancy from a longitudinal health insurance database of catastrophic illness in 2000 to 2008. All PC patients were categorized into 2 cohorts, namely, ADT cohort and non-ADT cohort, based on whether or not the patient receives ADT. The patients were followed up until the occurrence of AUR. Multivariate Cox proportional hazard regression and Kaplan–Meier analysis were performed. After a 12-year follow-up, the incidence rates of AUR were 12.49 and 9.86 per 1000 person-years in ADT and non-ADT cohorts, respectively. Compared with the non-ADT cohort, the ADT cohort had a 1.21-fold increase in AUR risk based on the adjusted model (95% CI = 1.03–1.43). In addition, PC patients receiving early ADT treatment within 6 months or receiving only luteinizing hormone-releasing hormone treatment also had significantly increased risk of AUR. ADT was positively associated with AUR risk. PC patients receiving ADT should be informed about the risks of bladder outlet obstruction and AUR, and they may benefit from screening for related risk factors. New guidelines and treatments should be proposed in the future to manage ADT-related lower urinary tract symptoms and reduce the risk of AUR.

## 1 Introduction

For patients with prostate cancer (PC), androgen deprivation therapy (ADT) has long been applied for primary and alternative therapy,^[1]^ which rapidly increased over the recent decades in the United States and worldwide due to newly developed high-potency androgen receptor inhibitors.^[2,3]^ Generally, approximately 40% to 45% of patients with localized or locally advanced PC received ADT.^[4,5]^ A large randomized clinical trial recently showed the significantly better overall and failure-free survival rate of patients with locally advanced or metastatic PC who received ADT combined with the administration of abiraterone acetate, a selective CYP17 inhibitor, than those of patients who received ADT alone.^[6]^

The risk of prostate cancer patients to show lower urinary tract symptoms(LUTS) and acute urinary retention (AUR) or other complications since the diagnosis (biopsy), early staged cancer and then later during disease.^[7–9]^ Growing evidence has shown the association between ADT and several health-related adverse effects, such as diabetes, cardiovascular disease, osteoporosis, and inflammatory bowel diseases.^[10,11]^ Recently, ADT is linked to overactive bladder symptoms with a time-dependent pattern due to the inhibitory role of androgen in male voiding modulation.^[12]^ A previous study showed that patients receiving ADT have higher scores in the American Urological Association benign prostatic hyperplasia (BPH) symptom index and a higher proportion of moderate to severe LUTS^[13]^ than those receiving other treatments, such as surgery or radiotherapy. The European Association of Urology guidelines warned that ADT is contraindicated in men with severe LUTS due to the risk of AUR,^[14]^ as a result of testicular deficiency caused by ADT, which emerged as a potential target by which metabolic syndrome may contribute to the worsening of LUTS.^[15]^ Given the limited evidence on the risk of AUR for ADT, we constructed a population-based study to clarify the correlation between ADT and AUR risk.

## 2 Materials and methods

### 2.1 Data source

We obtained 2 longitudinal health insurance databases (LHIDs) from the National Health Insurance Research Database (NHIRD) derived from the Taiwan National Health Insurance (TNHI) program by the Taiwan Bureau of National Health Insurance (TBNHI). One LHID included all catastrophic illness patients, and another included 1 million insurant randomly selected from the 1996 to 2000 Registry for Beneficiaries of the NHIRD. Thirty diseases fitted in the catastrophic illness categories, including malignancy, organ transplant, and mental diseases etc. These 2 databases are linked to each other according to the identification of the insurant. The TNHI program with a coverage rate is over 99% because all Taiwanese compulsorily join in this program. In accordance with the Personal Information Protection Act, the identification of insurant is recorded by TBNHI, and all researchers have to sign a written agreement for no intent to obtain personal information to protect the privacy of patients. This study was also approved by the Research Ethics Committee of China Medical University Hospital (CMUH104-REC2-115). According to NHIRD, the disease is identified based on the International Classification of Diseases, Ninth Revision, Clinical Modification.

### 2.2 Study participants

The participants were 24,464 male patients who were newly diagnosed with prostate malignancy (ICD-9-CM 185) from the LHID of catastrophic illness patients in 2000 to 2008. Patients who received ADT within 30 days after the date of prostate malignancy diagnosis were excluded. Patients were grouped into 2 cohorts, namely, the ADT cohort and the non-ADT cohort, based on whether or not they received ADT. The index date in the ADT cohort was defined by the date of ADT treatment. ADT patients with other malignancy diagnoses (ICD-9-CM 140–184 and 186–208) before the date of prostate malignancy diagnosis or with age <20 years, and the patients who received long-term Foley or cystostomy were excluded from the study. We also excluded patients with acute urine retention (AUR, ICD-9-CM 788.2) development within1 year after the index date to avoid reverse causation. The non-ADT cohort was randomly selected from prostate malignancy patients with frequency-matched criteria according to age strum (such as 20–24, 25–29, and 30–34), and the year of index date at a ratio 1:1 for the ADT cohort. Similar exclusion criteria were applied for non-ADT cohort.

### 2.3 End point, treatment, and baseline comorbidity

All patients were followed-up from the index date until the day of AUR occurrence. Those without AUR occurrence were followed up until the date of withdrawal from the NHI program or at the end of 2011. The ADT treatment consisted of bilateral simple orchiectomy and administration of luteinizing hormone-releasing hormone (LHRH), which was composed of leuprorelin (Anatomical Therapeutic Chemical code [ATC code)] L02AE02), goserelin (ATC code L02AE03), and triptorelin (ATC code L02AE04). Other treatments and medication included prostatectomy, radiotherapy, and administration of alpha blockers (ATC code C02CA) and 5α-reductase inhibitors (5ARIs, ATC code G04CB). Baseline histories of comorbidity (ICD-9-CM) included coronary artery disease (CAD, 410–414), stroke (430–438), diabetes (250), hypertension (401–405), hyperlipidemia (272), and lower leg fracture or surgery (820, 821, and 823, and ICD-9-CM operation code 81.51–81.54). For validity, patients with at last 3 medical visits were defined as patients, except lower leg fracture or surgery.

### 2.4 Statistical analysis

The differences in distribution of age group (< 65, 65–69, 70–74, 75–79, and 80+ years), treatment, treatment, and baseline comorbidity between the ADT and non-ADT cohort were tested by Chi-Squared test. The continuous variable was age, and the duration between the index date and prostate malignancy diagnosis date was presented as mean and standard deviation (SD). A t-test was performed to test the difference between the 2 cohorts. The incidence density rate (per 1000 person-years) was calculated using the number of cumulative AUR occurrence divided by the number of cumulative person-years during the study period in the 2 cohorts. Cox proportional hazard regression was used to assess the hazard ratio (HR) and 95% confidence intervals (CIs) of AUR in the ADT cohort relative to those of the non-ADT cohort. The model was adjusted for age, treatment, and all baseline comorbidity. According to the duration between the occurrence of prostate malignancy and index date, patients with the duration of less than or equal to 180 days were defined as the early treatment group, whereas those with the duration of more than 180 days were defined as the late treatment group. We also assessed the association between AUR and different ADT treatments combined with only orchiectomy, only LHRH, or both. The type of ADT treatment was defined before the end point. The interaction of ADT and age, comorbidity (including CAD, diabetes, stroke, hypertension, hyperlipidemia, and lower leg fracture or surgery), and other treatment and medication (including prostatectomy, radiotherapy, alpha blocker, and 5ARIs) were assessed by adding their product terms into the full model and the likelihood ratio test was used to test its significance. Relative excess risk due to interaction (RERI), proportion attributable to interaction (AP), and synergy index (S index) were calculated to estimate whether the interaction was on an additive scale. PERI or AP = 0 means no interaction; PERI or AP > 0 means positive interaction; and PERI or AP < 0 means negative interaction. S index = 1 means no interaction; S index > 1 means positive interaction; and S index <1 means negative interaction.^[16]^ The association between AUR and different ADT treatments timing, and AUR and different ADT type stratified by age were also estimated based on the interaction test. All statistical analyses were performed by SAS software version 9.4 (SAS Institute, Cary, NC). The statistical significance level was set at P < .05 by two-tailed testing.

## 3 Results

All 5574 ADT patients and 5574 matched comparisons were collected in this retrospective cohort study. In the ADT cohort, 71.3% of the patients were older than the 70 years, and the mean age was 73.8 years (SD = 8.20) (Table 1). Compared with the non-ADT cohort, the ADT cohort had less prevalence of CAD (37.1% vs 43.5%), diabetes (25.1% vs 27.1%), stroke (24.5% vs 28.4%), and hypertension (65.3% vs 67.5%). Except for radiotherapy treatment, ADT patients received less treatments, including prostatectomy, alpha blockers, and 5ARIs than the comparisons. In the ADT cohort, the duration between the occurrence of prostate malignancy and receiving ADT treatment was 0.73 years (SD = 1.48).

**Table 1:**
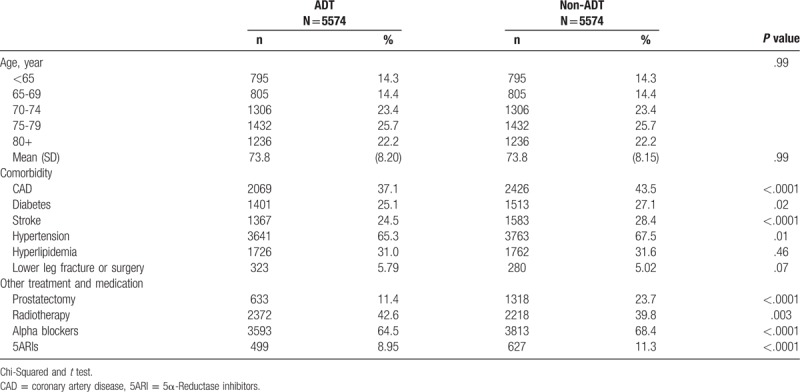
Distribution of age and comorbidities between prostate cancer patients with and without ADT treatment.

**Figure 1:**
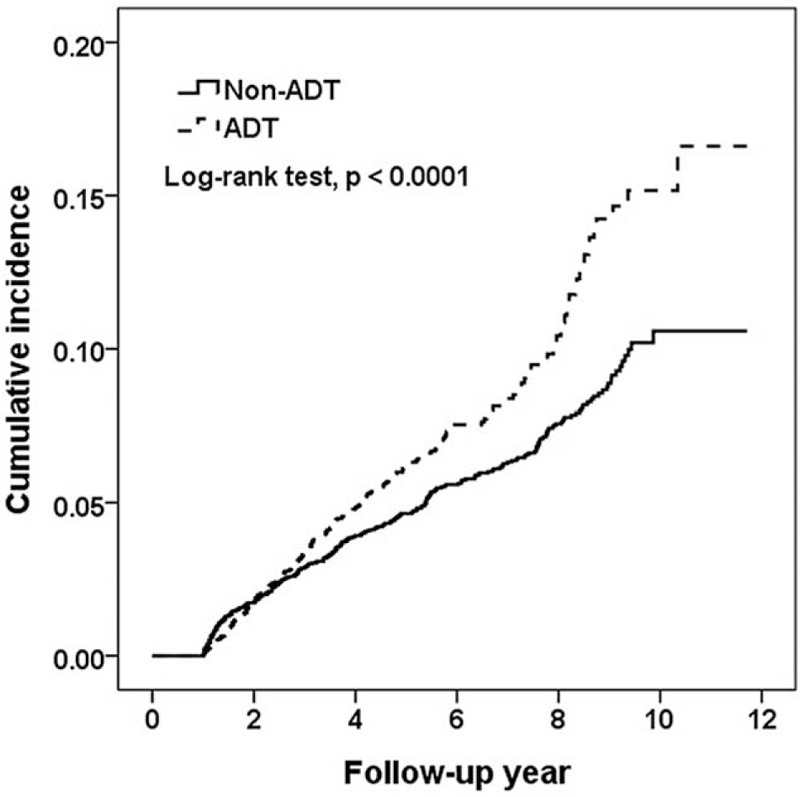
Cumulative incidence of acute urinary retention in non-ADT and ADT groups.

During the study period, 286 and 307 patients had AUR, and the incidence rates of AUR were 12.49 and 9.86 per 1000 person-years in the ADT and non-ADT cohorts, respectively (Fig. 1). Compared with the non-ADT cohort, the ADT cohort had a 1.35- and 1.21-fold AUR risk in crude and adjusted model (95% CI = 1.14–1.59 and 1.03–1.43, respectively) (Table 2). Based on the duration between the occurrence of prostate malignancy and receiving ADT treatment, over 75% of patients receiving early treatment within 6 months had high AUR incidence (13.19 per 1000 person-years), followed by late treatment (9.54 per 1000 person-years). Compared with the non-ADT cohort, ADT patients with early treatment had significantly high AUR risk (HR = 1.24, 95% CI = 1.04–1.48) after adjusting for potential risk factors. In addition, approximately 67% of patients were receiving only LHRH treatment, 24% only orchiectomy, and 9% both treatments. Patients receiving only LHRH treatment had a 1.23-fold increase in AUR risk (95% CI = 1.03–1.49) compared with non-ADT patients.

**Table 2:**
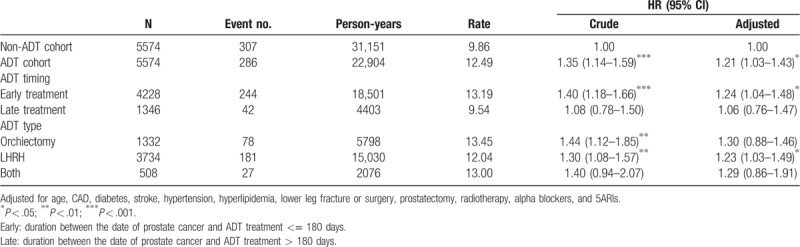
Associations between hazard ratio of AUR and receiving ADT therapy stratified by timing and types.

Table 3 was presented the AUR risk stratified by age, comorbidity, and other treatment and medication. The result showed that ADT users compared with non-user at the age < 80 years had a significantly higher AUR risk than those at age 80+ years (HR = 1.36 vs 0.81, 95% CI = 1.12–1.64 vs 0.57–1.14, interaction *P* = .005) in adjusted model. PERI, AP, and S-index were −0.54, −0.45, and 0.27, respectively, indicating that there exists a negative interaction. In different comorbidity, and other treatment and medication, there were the similar trend when ADT users compared with non-user (all interaction *P* > .05).

**Table 3:**
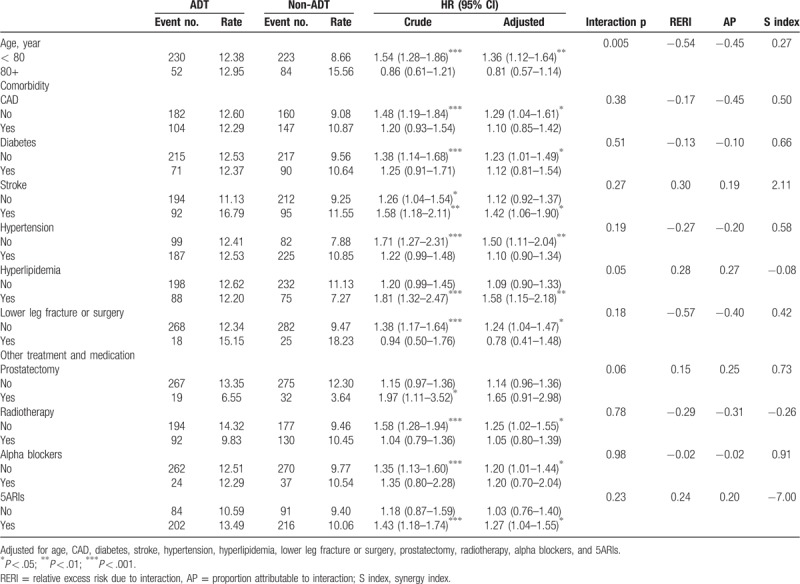
Associations between hazard ratio of AUR stratified by age, comorbidities, and medical treatment.

Table 4 was showed the association between AUR risk and different ADT treatments timing, and AUR and different ADT type stratified by age. In those age < 80 years, ADT users in early treatment had a 1.39-fold AUR risk (95% CI = 1.14–1.69), and ADT users combined only LHRH or both (LHRH and orchiectomy) had a 1.35- and 1.57-fold AUR risk (95% CI = 1.09–1.68, and 1.05–2.35), respectively, compared with non-users in adjusted model. But in those age 80+ years, there were no significantly difference of AUR incidence between ADT users and non-users.

**Table 4:**
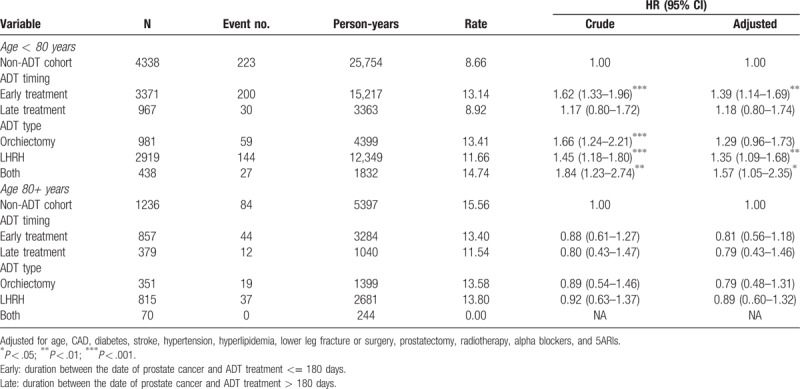
Associations between hazard ratio of AUR stratified by timing and types in different age group.

## 4 Discussion

This study is the first large-scale, long-term follow-up and population-based study that investigated the correlation between ADT and AUR risk. This correlation may cause physical and mental suffering and interfere with compliance during the treatment course for men with PC. We showed that PC patients receiving ADT had significantly increased risk of AUR after adjusting for possible confounders, especially for those aged less than 80, who started ADT within 6 months and those receiving only LHRH.

Men with PC suffer from AUR due to the concurrent benign prostatic enlargement or following radiotherapy.^[17]^ However, the mechanisms and associations between ADT and AUR are still controversial. Recently, Li et al conduct a population based study and resulted that ADT for men with prostate cancer cause significantly higher overactive bladder symptoms, which may due to the inhibitory role of androgen in voiding function modulation.^[12]^ Emerging evidence indicated that decreased testosterone levels during ADT may negatively influence body composition, mental health, fatigue, and quality of life, as well as several adverse effects such as increased cardiovascular and metabolic risk and urinary problems, and age accounts for a significantly independent predictor for AUR in patients received ADT.^[18,19]^ The administration of GnRH agonists, but not orchiectomy, is associated with a significantly increased risk of CAD, AMI, and SCD,^[20]^ which may increase the risk of AUR., A supervised exercise intervention would improve inflammatory and lipid status as well as cardiorespiratory capacity, reduce abdominal fat mass, and improve the levels of self -reported QoL and LUTS.^[21]^

ADT may be related to metabolic syndrome, which aggravates male LUTS through inflammatory processes is linked to bladder outlet obstruction, and might also be associated with episodes of AUR.^[22,23]^ The elevation of follicle-stimulating hormone during ADT treatment contributes to the development of atherosclerosis and insulin resistance,^[24]^ 2 important factors that exacerbate male bladder outlet obstruction. ADT-related cognitive dysfunction might be another possible issue associated with AUR.^[25,26]^ One recent study proposed the association between urinary dysfunction and cognitive impairment in patients with Parkinson's disease.^[27]^ In another study, patients with multiple sclerosis experienced incomplete bladder emptying due to cognitive dysfunction; this condition required catheterization to improve quality of life.^[28]^ Penile atrophy was another possible issue after long-term ADT, which poses urination difficult for some men and is associated with AUR and significant decrease in the quality of life.^[29]^

Previous studies showed that triptorelin, a gonadotropin-releasing hormone agonist, improved clinically significant LUTS in patients under ADT treatment; this effect was especially perceived within the first 12 to 24 weeks, but failed to show the association with AUR.^[30,31]^ We thought that was a short-term prostate-receptor effect similar to the effect of alpha blockers, because the effect of IPSS improvement mediated by prostate volume reduction will be significant not shorter than 6 months.^[32]^ Instead, metabolic effect will persist after long-term use and will increase the risk of AUR then after. Within a short period of time from the beginning, ADT had significant impact on the physical and sexual aspects of the patients, which were 2 mainstays for quality of life and were independent of confounders like cancer diagnosis or surgical treatment.^[33]^ As quality of life is one of the main decisive roles for PC men receiving ADT, careful counseling for the clinical outcomes and adverse effects would be necessary.^[34]^ Recommendations including exercise, nutrition adjustment, or medical algorithm that prevent the risk of AUR may potentially improve outcomes and compliance for ADT.^[33]^

Some limitations needed to be discussed in the present results. First, we lack some clinical data such as cancer stage and grade. Among early-stage cancer patients, the likelihood of finding a man suffering AUR may be higher because they are examined longer during follow-up. Second, data on prostate volume, which contribute important information to the occurrence of LUTS and AUR, were scarce. Patients with large prostate volume or with PC often come concurrently due to the biological similarity and diagnosis selection of the urological society guideline.^[17]^ Third, surveillance bias was another potential study limitation; because PC patients receiving ADT treatment were being visited more frequently than those without ADT, physicians were more likely to detect AUR symptoms among these patients. Regardless of these limitations, the present study collected total incidence cases of PC patients through the nationwide LHID-CIP database and constructed a longitudinal study design to explore the association between ADT and AUR risk.

In conclusion, PC patients receiving ADT had significantly increased risk of AUR, especially for those who were younger (less than 80 years old), started ADT within 6 months and those receiving only LHRH treatment. We suggested that PC patients receiving ADT should be informed about the risk of bladder outlet obstruction and AUR, and they may benefit from screening for the modifiable risk factors and from regular, periodic assessments. New guidelines and treatments should be proposed in the future to manage ADT-related LUTS and reduce the risk of AUR.

## Author contributions

**Conceptualization:** Teng-Kai Yang, Chia-Chang Wu, Chao-Hsiang Chang, Chao-Yuan Huang, Chi-Jung Chung.

**Data curation:** Chao-Hsiang Chang, Chao-Yuan Huang, Chih-Hsin Muo, Chi-Jung Chung.

**Formal analysis:** Chih-Hsin Muo, Chi-Jung Chung.

**Writing draft:** Teng-Kai Yang, Chia-Chang Wu, Chih-Hsin Muo, Chi-Jung Chung.
